# Improving Detection of Arrhythmia Drug-Drug Interactions in Pharmacovigilance Data through the Implementation of Similarity-Based Modeling

**DOI:** 10.1371/journal.pone.0129974

**Published:** 2015-06-12

**Authors:** Santiago Vilar, Tal Lorberbaum, George Hripcsak, Nicholas P. Tatonetti

**Affiliations:** 1 Department of Biomedical Informatics, Columbia University, New York, NY, United States of America; 2 Department of Systems Biology, Columbia University, New York, NY, United States of America; 3 Observational Health Data Sciences and Informatics (OHDSI), New York, NY, United States of America; 4 Department of Medicine, Columbia University, New York, NY, United States of America; National Chiao Tung University, TAIWAN

## Abstract

Identification of Drug-Drug Interactions (DDIs) is a significant challenge during drug development and clinical practice. DDIs are responsible for many adverse drug effects (ADEs), decreasing patient quality of life and causing higher care expenses. DDIs are not systematically evaluated in pre-clinical or clinical trials and so the FDA U. S. Food and Drug Administration relies on post-marketing surveillance to monitor patient safety. However, existing pharmacovigilance algorithms show poor performance for detecting DDIs exhibiting prohibitively high false positive rates. Alternatively, methods based on chemical structure and pharmacological similarity have shown promise in adverse drug event detection. We hypothesize that the use of chemical biology data in a post hoc analysis of pharmacovigilance results will significantly improve the detection of dangerous interactions. Our model integrates a reference standard of DDIs known to cause arrhythmias with drug similarity data. To compare similarity between drugs we used chemical structure (both 2D and 3D molecular structure), adverse drug side effects, chemogenomic targets, drug indication classes, and known drug-drug interactions. We evaluated the method on external reference standards. Our results showed an enhancement of sensitivity, specificity and precision in different top positions with the use of similarity measures to rank the candidates extracted from pharmacovigilance data. For the top 100 DDI candidates, similarity-based modeling yielded close to twofold precision enhancement compared to the proportional reporting ratio (PRR). Moreover, the method helps in the DDI decision making through the identification of the DDI in the reference standard that generated the candidate.

## Introduction

Medication co-administration can alter the pharmacokinetic or pharmacodynamic profiles of the drugs being prescribed. Drug-Drug Interactions (DDIs) occur when the effect of one drug is altered by the co-administration of another drug. This change in the effect can lead to the development of clinically important adverse events. In fact, a significant amount of the adverse effects caused by drugs in the patients are due to the administration of multiple medications [1–3]. As an example of DDIs, some macrolides, such as erythromycin, inhibit the metabolism and the elimination of warfarin [4]. This fact could cause an increased effect of warfarin with the consequent risk due to its anticoagulant properties. Another example is the combination of simvastatin and posaconazole, associated with a risk of myopathy and rhabdomyolysis due to increased statin plasma concentrations [5].

Pharmacovigilance focuses on the collection, monitoring and evaluation of adverse events caused by drugs and other biological products in the pharmaceutical market. Pharmacovigilance agencies, such as the FDA U. S. Food and Drug Administration, are interested in the use of post-marketing data to analyze possible adverse drug effects (ADEs) and possible DDIs that cause higher impact in ADE development. However, improvements in the current approaches are still needed to help in the early detection of DDIs.

Recently, a number of computational methods have been successfully applied to predict DDIs. Among them, cheminformatic methodologies, such as protein-structure-based and ligand-based methods, have been used in the detection of DDIs. Cheminformatics provides a useful approach through the use of 2D/3D QSAR (quantitative structure-activity relationships) [6–8], homology modeling [9] and molecular docking [10]. These methods can infer similarity between sets of drugs [11–13] and study possible interactions with pharmacodynamics or pharmacokinetic targets. In previous work, we have leveraged cheminformatics to construct general models of DDIs [11, 12].

On the other hand, scientific literature and pharmacovigilance databases are additional sources with important implications in DDI discovery [3, 14]. Percha *et al*. [15] mined the scientific literature to detect DDIs through the extraction of gene-drug relationships. Mining electronic health records (EHRs) or the FDA’s Adverse Event Reporting System (FAERS) [16] is an alternative for the discovery of DDIs [1, 17]. In fact, Tatonetti *et al*. recently provided an important source of DDI candidates, the TWOSIDES database [18], through mining FAERS. However, analysis of pharmacovigilance data is still very challenging and rampant confounding leads to high false positive rates. Alternatively, cheminformatic methods can be applied to rank the DDI candidates extracted from a pharmacovigilance study. These methods offer the possibility to study the final candidates from the point of view of the molecular structure, pharmacological action or adverse effects comparison. Similarity-based methods were useful to rank drug candidates extracted from pharmacovigilance data mining that produce some adverse events, such as rhabdomyolysis and pancreatitis [19, 20].

**Fig 1:**
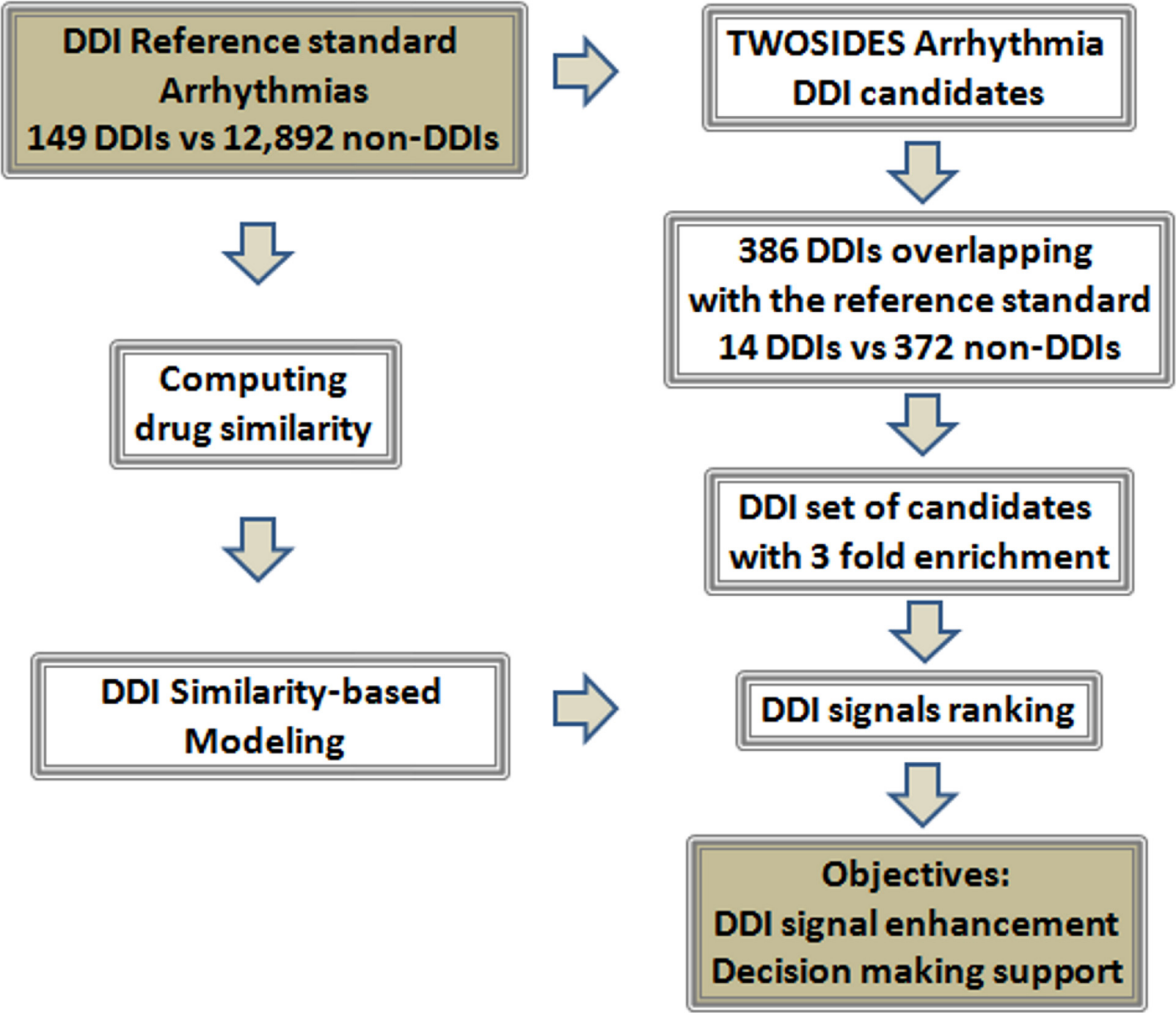
Flowchart with the different steps implicated in the study.

In this paper, we systematically apply six different similarity-based techniques to evaluate drug interaction hypotheses mined from pharmacovigilance data. The objective of the current study is to improve the detection of DDIs in the TWOSIDES database using methodologies we recently developed based on the application of similarity-based modeling (see Fig 1). When applied to the TWOSIDES database a reference standard of DDIs that produce arrhythmia, we measured: 1) enrichment factor provided by TWOSIDES, and 2) performance when we rank the set of DDI candidates using proportional reporting ratio (PRR), *p*-values, and different similarity-based models. As is demonstrated by our results, the implementation of cheminformatic models in pharmacovigilance data is useful in DDI signal detection and decision making process.

## Methods

### DDI reference standard

We collected a reference standard with 149 DDIs present in the intersection of both DrugBank [21] and Veterans Association Hospital database [22]. The collected DDIs produced the effect of arrhythmias and related terms, such as QT prolongation or increased heart rhythm. In our reference standard there are DDIs with different levels of documentation, from “well established through controlled studies” to “theoretical interactions but pharmacological reasons lead clinicians to recognize the possible interaction”. The 149 DDI pairs comprised 162 drugs and were included in a 162×162 drug-drug matrix called M_1_ (13,041 total number of possible interactions). We codified the 149 reference standard DDIs in M_1_ with value 1 in each respective cell, and the non-DDIs with value 0 (see S1 and S2 Tables).

### Drug similarity-based calculation

**Fig 2:**
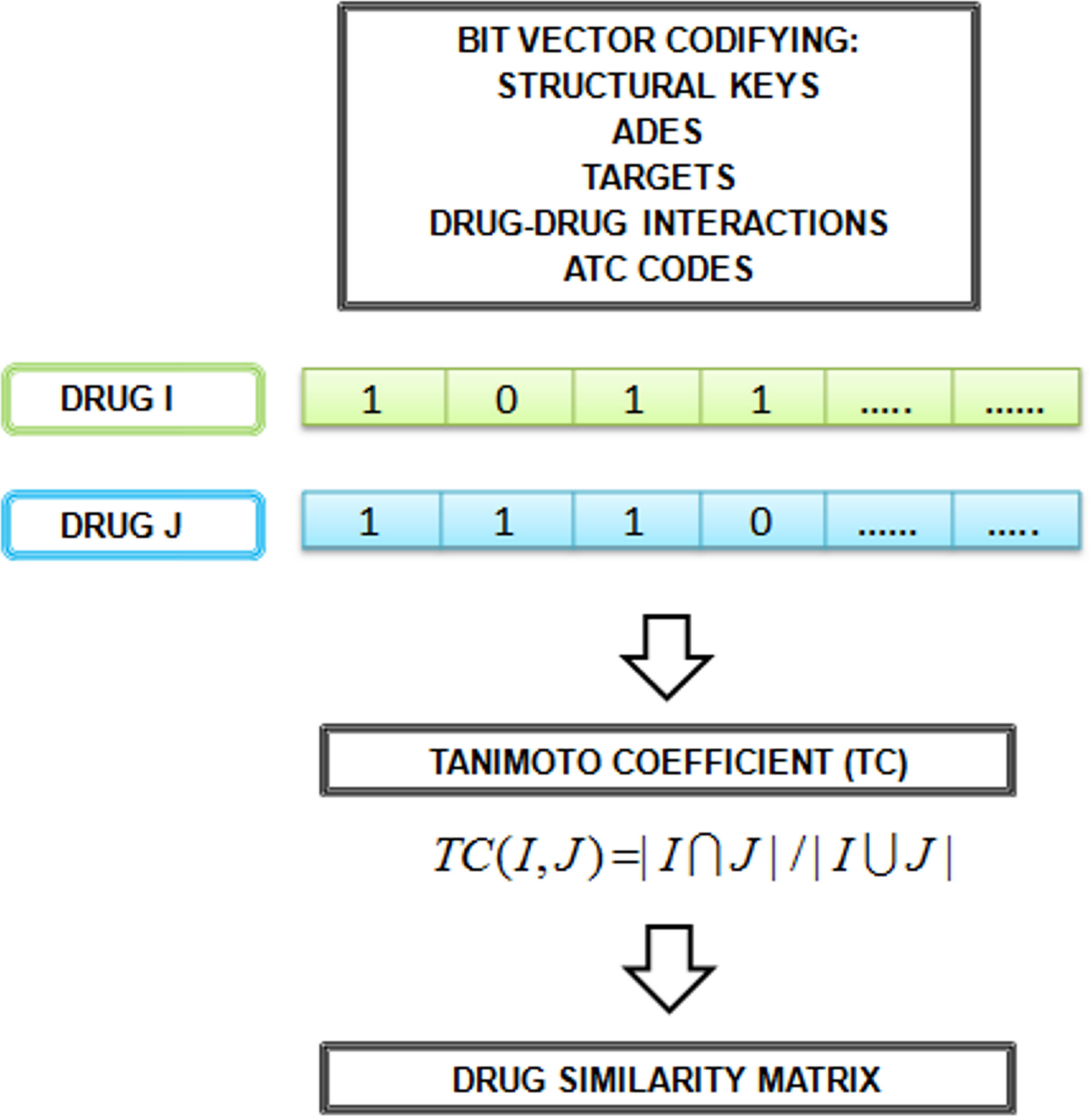
Flowchart including the steps implicated in the calculation of different similarity measures. Drugs were represented as fingerprints, i.e. bit vector codifying the presence or absence (1, 0) of structural keys, adverse effects, targets, drug-drug interactions or ATC codes. The Tanimoto coefficient (Tc) between all the fingerprint pairs is calculated and placed in a drug-drug similarity matrix (M_2_). Different M_2_ matrices are calculated weighted with the different similarity measures.

To calculate drug similarity we used different measures. Fig 2 shows the workflow used to calculate some similarity measures. A detailed explanation about the construction of drug similarity-based models can be found in previous publications [11, 12]. Different drug similarity matrices (M_2_) were generated at this step (the data is provided in S3 Table).

#### 2D molecular structure drug similarity (matrix M_2a_)

We calculated MACCS fingerprints for all the 162 drugs in our reference standard. MACCS represents the 2D molecular structure as a vector that codifies the presence or absence (1 and 0 codes) of different structural keys or sub-fragments. A detailed description of the fingerprint calculation can be found in previous publications [11, 23]. We compared pairs of MACCS fingerprints using the Tanimoto coefficient (Tc). The Tc is the ratio between the number of features (structural keys in our case) in the intersection and the union of two fingerprints. The Tc ranges from 0 to 1, which means minimum and maximum similarity respectively. Once we calculated the Tc for all the drug pairs, we constructed a 162×162 drug similarity matrix (M_2a_). In each cell of the matrix we placed the Tc for the drug pair (see Fig 2).

#### 3D molecular structure drug similarity (matrix M_2b_)

We downloaded from DrugBank [21] the isomeric SMILES codes of the 162 drugs in our reference standard. Isomeric SMILES codes provide information about the chemical structure but also allow the specification of the configuration of chiral centers. We pre-processed the database using the LigPrep module in the Schrödinger 2011 package [24]. Through this process, when there are non-specified chiral centers in some drugs, a maximum of three enantiomers was generated. We performed Monte Carlo Multiple Minimum (MCMM) conformational analysis calculations using Macromodel [24] to determine the most stable 3D molecular structure for each drug. We retained the structure with the minimum potential energy OPLS_2005 as a drug-template for the next shape screening step. Using these 3D drug structure templates generated through MCMM, we performed shape screening calculations with Phase module [24] to identify similar molecules to the templates. The calculation performed a flexible alignment between the 3D conformations of drug *i* with the rigid 3D structure template of drug *j* and identified similarities between pair of drugs based on similar 3D distribution of pharmacophoric features. We calculated a 3D similarity score (Phase Sim property) that ranges from 0 to 1 indicating minimum and maximum similarity respectively. 3D scores between all the pairs (162×162) were integrated in the 3D similarity matrix M_2b_. A more detailed explanation about 3D calculation parameters can be found in previous references [24, 25].

#### Adverse drug effect profile fingerprint (ADEPF) similarity (matrix M_2c_)

Adverse effects were collected from SIDER database [26], an open resource of drugs and related side effects extracted from public documentation and package inserts. The adverse effects for each drug were represented as fingerprints, i.e. bit vector codifying the presence or absence (1, 0) of adverse effects. As explained previously in the study (*s*ee 2D molecular structure drug similarity section), we calculated the Tc between all the fingerprint pairs and constructed the matrix M_2c_ with ADE similarity information between all the drugs (see Fig 2).

#### Target profile fingerprint (TPF) similarity (matrix M_2d_)

We collected the targets for each drug using DrugBank [21]. We integrated the datasets with information about targets, enzymes, transporters and carriers. The same target protein but from different organisms was considered as a unique case. As we explained previously, we represented targets in each position of a fingerprint and then we calculated the Tc between all the fingerprint pairs. In the final step, we constructed the matrix M_2d_ weighted with target information including in each cell the Tc between the corresponding drug pair.

#### Drug-drug interaction profile fingerprint (DDIPF) similarity (matrix M_2e_)

The concept of drug-drug interaction profile fingerprints was introduced in a previous study [12]. Each drug was represented as a vector that codifies the presence (code 1) or the absence (code 0) of the different drug-drug interactions, i.e., in our case we constructed DDIPFs with drug interaction information from DrugBank [21]. Tc comparing the DDIPFs was included in the matrix M_2e_ (see Fig 2).

#### ATC-codes fingerprint similarity (matrix M_2f_)

We used the Anatomical Therapeutic Chemical (ATC) Classification System [27] to calculate similarities between drugs. We considered four levels in the ATC codes, involving information in different categories: location (organ or system), therapeutic, pharmacological, and chemical properties. The different groups in each level were represented as vector positions and Tc was calculated between all the ATC-code fingerprint pairs. As previously, we constructed the matrix M_2f_ with ATC-codes similarity.

### Calculation of DDI candidates

The method to generate the new set of DDI candidates has been recently described by our research group [11, 12, 25, 28]. Through this step a new DDI matrix (M_3_) is calculated with the DDI score for each pair of drugs in each respective cell. It is worth noting that diagonal values in the initial matrices M_2_ are set 0 not representing similarity of a drug with itself. The final DDI score provided by M_3_ is based on a leave-one-out process. To generate the final matrix M_3_ with all the drug pairs DDI candidates we multiplied M_1_ by M_2_ retaining only in each cell the highest value in the addition-array. Although in each cell all the scores against the set of reference standard DDIs are generated (the matrix product generated in each cell the addition of the different scores), only the highest score is retained to represent the maximum similarity against the well-known DDIs. The resultant matrix is not symmetric (similarity is implemented in both branches of the drug-drug pair), for which a symmetric transformation is carried out retaining the maximum value in each symmetric cell. That way, each cell in the final M_3_ matrix represents the drug pair DDI candidate with the maximum similarity score regarding to a DDI drug pair deemed as true positive in our reference standard. DDIs from the M_3_ matrix are listed with their corresponding similarity scores (these data is provided in S4 Table). DDIs belonging to the matrix diagonal and representing drugs interacting with themselves are eliminated. Although our models are based on the maximum similarity score, the method allows the implementation of alternative algorithms.

### Pharmacovigilance data: TWOSIDES database

We downloaded the TWOSIDES database [18], a data source of DDIs extracted from mining FAERS [16]. We collected 13,105 DDIs related to the terms arrhythmia and bradyarrhythmia with proportional reporting ratio (PRR)>1 and *p*-value <.05. These data were mapped to our initial DDI reference standard to find the DDIs in common. We retained 386 DDIs present in both databases: 14 positives and 372 negatives (see S5 Table). The subset of final DDIs was sorted by PRR and *p*-value (provided by TWOSIDES) and by the different similarity-based models.

### Combination of similarity-based modeling

We constructed different complex models combining the M_3_ similarity-based scorings for the 386 cases analyzed in TWOSIDES. We used Principal Component Analysis (PCA) and Linear Discriminant Analysis (LDA). Through PCA the six M_3_ scorings (2D, 3D, ADEPF, TPF, DDIPF and ATC) were transformed into a unique component explaining the 66.4% of the variance (Factor loadings were: 2D MACCS = -0.87, 3D = -0.85, ADEPF = -0.85, TPF = -0.74, DDIPF = -0.84, ATC = -0.73). The percentage of the variance explained by each additional factor is provided in S1 Fig On the other hand, we trained a LDA model with 14 positives and 372 negative cases. Five variables were introduced in the model using the forward-stepwise method: 2D, 3D, TPF, DDIPF and ATC scores. Statistical quality of the model was assessed through parameters such as Wilks’ statistic (*U* = 0.84), Fisher ratio (*F* (5, 380) = 14.8) and the significance level (p<.0001). S1 Fig also provides the AUROC results of LDA including from 1 to 5 variables in the model.

### Assessment of the performances

We measured the enrichment factor (EF) detected in TWOSIDES as the ratio between the prevalence detected in TWOSIDES and the prevalence in the initial reference standard. Prevalence is defined as the proportion of known well-established DDIs between all the DDI candidate cases. We also ranked the DDIs according to proportional reporting ratio (PRR) and *p*-value, provided by the TWOSIDES, and according to our different similarity DDI models and assessed the precision in different top positions. Precision or positive predictive value was calculated as the ratio between true positives and all positive cases, true positives plus false positives (Precision = TP/TP+FP). For the comparison of the performances we also used areas under the receiver operating characteristic curves (in the manuscript this partial AUROC for the subset of TWOSIDES DDI candidates is defined as pAUROC). If the area under the curve is 0.5 the classifier is random whereas a perfect classifier will yield an area of 1. ROC curves were also plotted showing the true positive fraction (sensitivity) against the false positive fraction (1-specificity). We performed an external evaluation using reference standard data sources, such as Drugdex (Micromedex) [29] and Drugs.com [30], to deem the rest of 372 candidates as positive and negative cases.

## Results

### Performance in TWOSIDES using the initial reference standard

**Fig 3:**
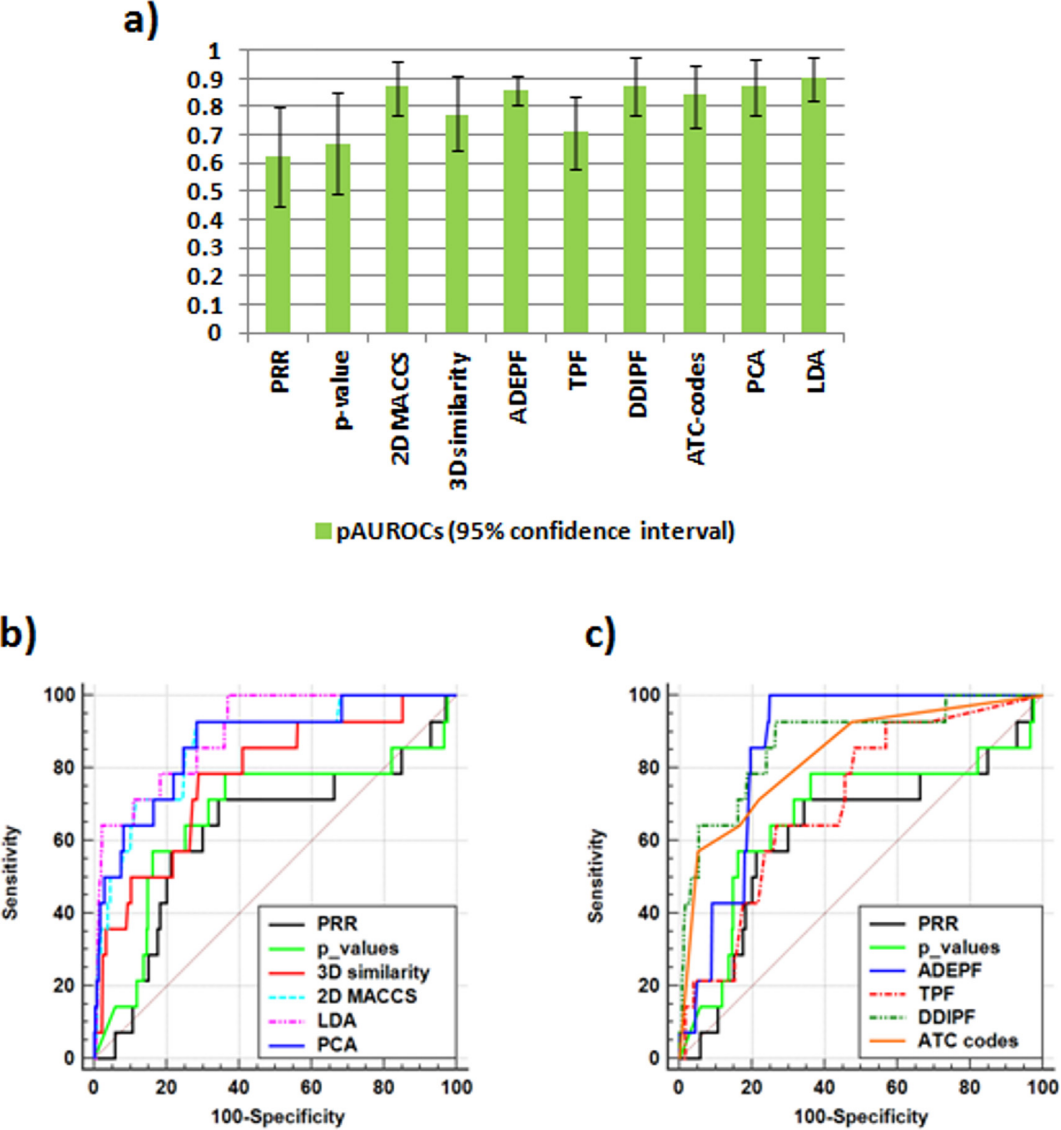
ROC results using different methods to rank the 386 TWOSIDES candidates: PRR (Proportional Reporting Ratio), *p*-values, 2D structural similarity (MACCS), 3D structural similarity, ADEPF (Adverse Drug Effect Profile Fingerprint), TPF (Target Profile Fingerprint), DDIPF (Drug-Drug Interaction Profile Fingerprint), ATC-code fingerprint, PCA (Principal Component Analysis) and LDA (Linear Discriminant Analysis). Panel (a) shows pAUROCs with 95% confidence intervals. Panels (b) and (c) show the ROC curves for the different methods.

We mapped our initial DDI reference standard (149 positive and 12,892 negative cases) to the arrhythmia DDI candidates extracted from TWOSIDES database to find DDIs in common. From TWOSIDES we identified 14 positive cases and 372 negatives, a 3-fold enrichment factor (*p* = .0003) (see Fig 1). We ranked the subset of DDI candidates obtaining an area under the ROC curve of 0.62 and 0.67 using proportional reporting ratio (PRR) and *p*-values as scorings (we defined this partial AUROC as pAUROC; see Fig 3).

### Application of similarity-based modeling in DDI signals ranking

As an alternative system to PRR and *p*-values, we ranked the subset of 386 DDI candidates extracted from TWOSIDES using the different similarity-based models. Fig 3 shows the pAUROC results and ROC curves for the different ranking methods used to sort the candidates, including PRR, *p*-values, (data shown previously) and all the similarity-based models using 2D and 3D similarity, ADEPF, TPF, DDIPF and ADE-codes similarities (pAUROC values, 95% confidence intervals and significance statistics are shown in S6 Table). The score provided by the models for each DDI is based on a leave-one-out procedure. Application of similarity models offered better results in the ranking process.

We constructed more complex models combining the different individual similarity scores through unsupervised methods, such as Principal Component Analysis (PCA), and supervised methods, such as Linear Discriminant Analysis (LDA) (see Fig 3). In PCA we combined the six similarity scorings (2D, 3D, ADEPF, TPF, DDIPF and ATC) in a simple component-scoring. Using LDA, five individual scores (2D, 3D, TPF, DDIPF and ATC) were introduced in the final model. Detailed description of the parameters for PCA and LDA are provided in the Methods section. The PCA and LDA models showed pAUROCs of 0.87 an 0.90 respectively.

### Assessing performance using alternative reference standards

In the set of 386 TWOSIDES DDI candidates, 372 were considered negative cases or non-DDIs according to our initial reference standard. However, as our reference standard may be incomplete, we assessed the detection of DDIs against two additional reference sources: Drugdex (Micromedex) [29] and Drugs.com [30].

#### Assessment of DDIs using Drugdex

We labeled the remaining 372 DDIs as true positives (TP) or false positives (FP) whether the interactions causing arrhythmias are described in Drugdex (Micromedex) or not. We considered different levels of knowledge to deem the interactions as positives: (Set 1) well-established interactions, probable, and theoretical are included (we found 164 TP and 208 FP); (Set 2) well-established interactions and probable are included (49 TP and 323 FP); (Set 3) only considered well-established interactions (10 TP and 362 FP). The pAUROC results are shown in Table 1. Our individual six similarity models, along with PCA and LDA models, performed well in set 1 whereas in set 2 and 3 the performance was poor. Only ATC-codes and LDA model showed some predictive power in set 3.

**Table 1:** pAUROCs using different methods to rank the DDIs. Drugdex (sets 1–3) and Drugs.com (sets 4–5) were used as reference standards.

pAUROCs (95% confidence interval)					
Scoring method	Drugdex Set 1 [164 TP and 208 FP]	Drugdex Set 2 [49 TP and 323 FP]	Drugdex Set 3 [10 TP and 362 FP]	Drugs.com Set 4 [231 TP and 141 FP]	Drugs.com Set 5 [87 TP and 285 FP]
**PRR**	0.53 (0.471 to 0.589)	0.56 (0.481 to 0.639)	0.55 (0.418 to 0.689)	0.52 (0.458 to 0.579)	0.58 (0.509 to 0.647)
***p*-value**	0.52 (0.458 to 0.576)	0.54 (0.459 to 0.616)	0.50 (0.338 to 0.669)	0.51 (0.454 to 0.574)	0.57 (0.501 to 0.638)
**2D MACCS**	0.66 (0.612 to 0.710)	0.51 (0.419 to 0.605)	0.53 (0.315 to 0.741)	0.61 (0.548 to 0.662)	0.65 (0.587 to 0.717)
**3D similarity**	0.68 (0.623 to 0.734)	0.43 (0.334 to 0.514)	0.34 (0.145 to 0.530)	0.68 (0.623 to 0.731)	0.67 (0.605 to 0.739)
**ADEPF**	0.68 (0.628 to 0.738)	0.52 (0.435 to 0.604)	0.46 (0.311 to 0.591)	0.62 (0.561 to 0.675)	0.65 (0.583 to 0.716)
**TPF**	0.70 (0.649 to 0.754)	0.54 (0.465 to 0.621)	0.47 (0.328 to 0.592)	0.65 (0.595 to 0.708)	0.67 (0.603 to 0.725)
**DDIPF**	0.75 (0.699 to 0.800)	0.55 (0.467 to 0.639)	0.55 (0.349 to 0.751)	0.69 (0.641 to 0.748)	0.67 (0.602 to 0.731)
**ATC codes**	0.69 (0.642 to 0.743)	0.56 (0.485 to 0.644)	0.66 (0.481 to 0.834)	0.61 (0.560 to 0.663)	0.68 (0.619 to 0.743)
**PCA**	0.75 (0.700 to 0.805)	0.53 (0.440 to 0.620)	0.51 (0.319 to 0.691)	0.69 (0.632 to 0.739)	0.71 (0.644 to 0.772)
**LDA**	0.73 (0.680 to 0.782)	0.61 (0.535 to 0.686)	0.73 (0.573 to 0.877)	0.63 (0.575 to 0.687)	0.66 (0.588 to 0.724)

In set 1, interactions well-established, probable and theoretical are considered true positives (TP). In set 2, interactions well-established and probable are considered TP. In set 3, only interactions well-established are considered TP. Set 4 included interactions with high and moderate clinical significance as TP. Set 5 included only highly clinically significant interactions as TP.

#### Assessment of DDIs using Drugs.com

**Fig 4:**
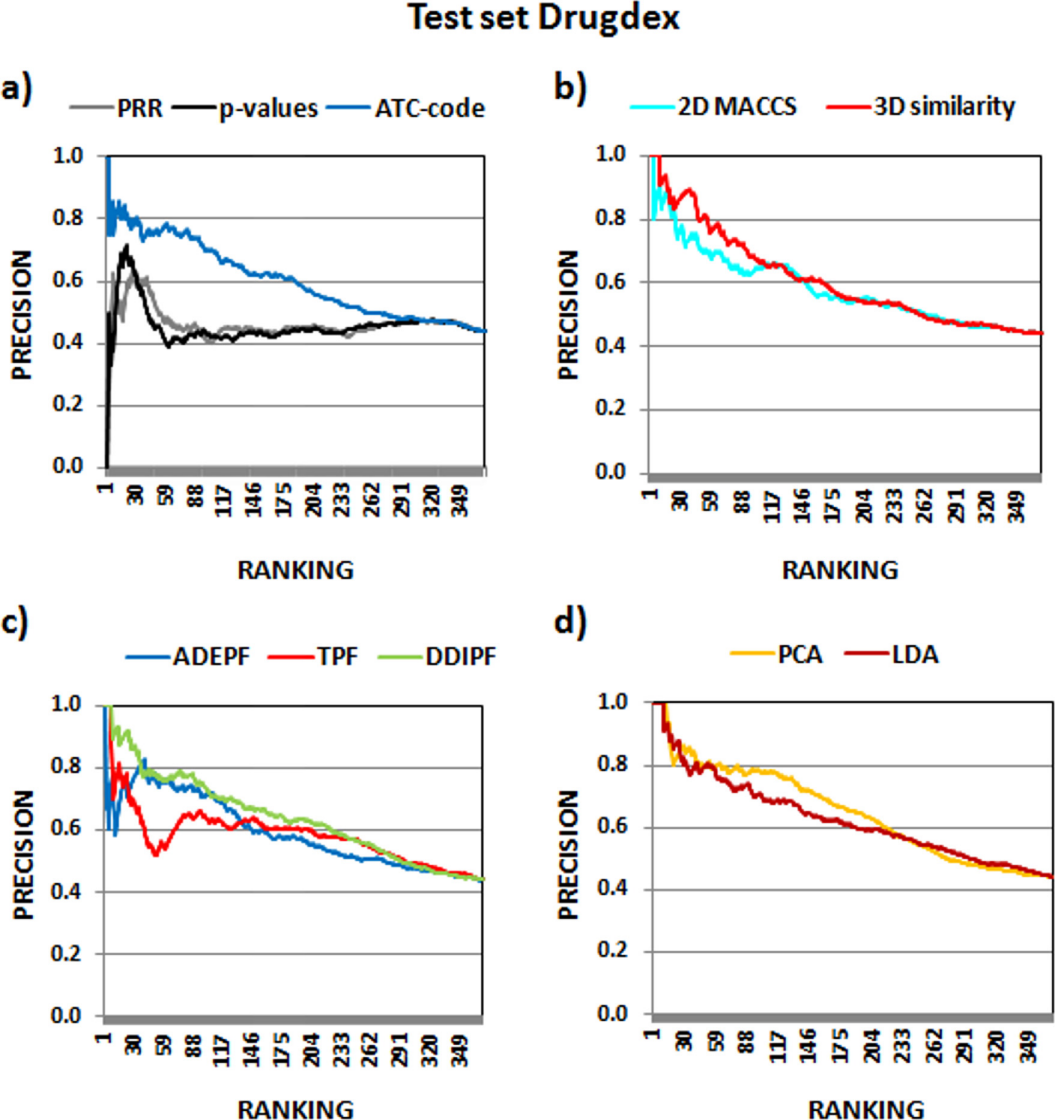
Precision of the different methods in test set 1 with all the interactions described in the reference standard Drugdex (interactions well_established+probable+theoretical).

**Fig 5:**
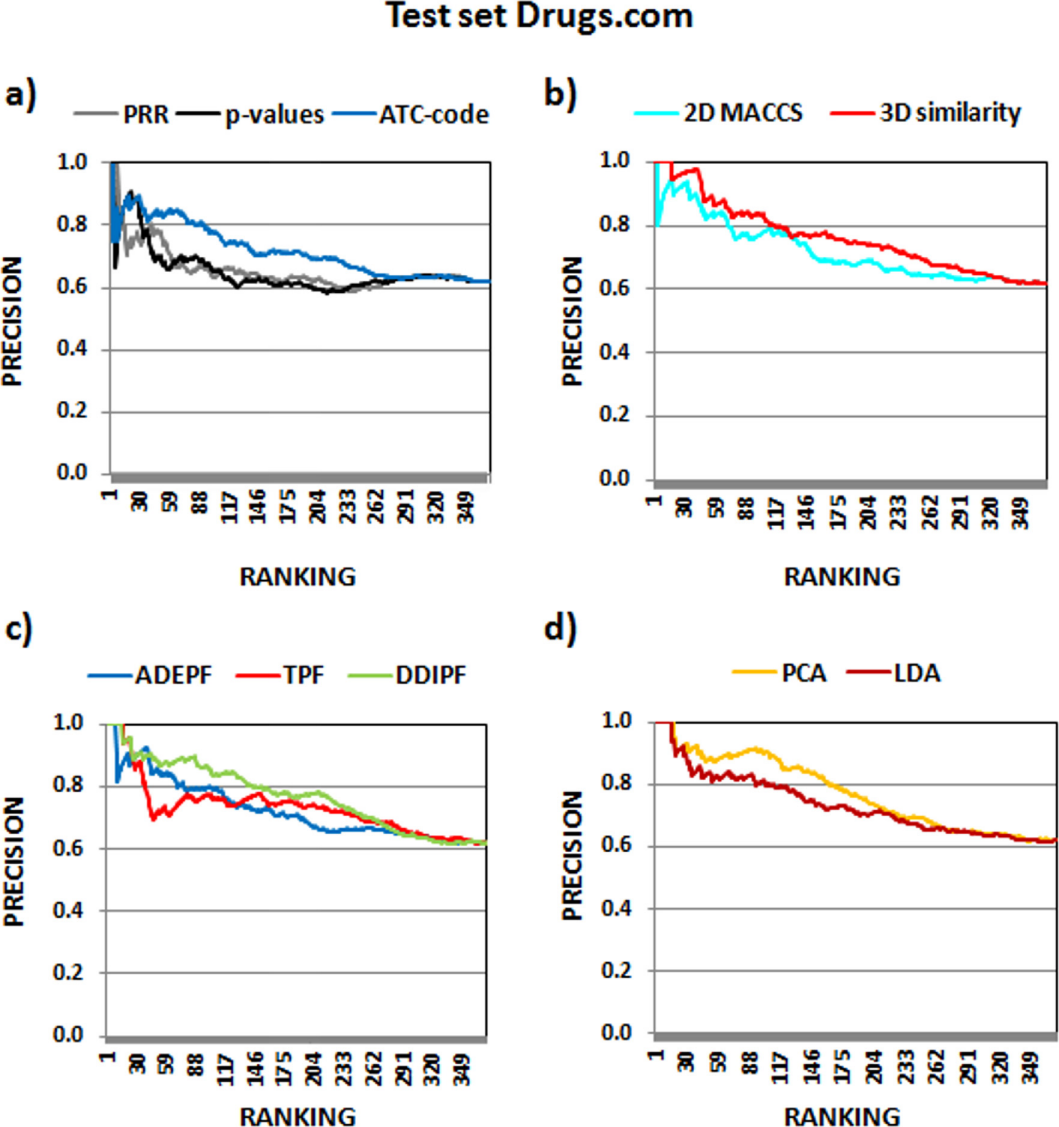
Precision of the different methods in test set 4 with all the interactions described in the reference standard Drugs.com (high and moderate clinically significant interactions).

The same 372 DDIs were analyzed using Drugs.com as a reference standard. Two sets of DDIs were considered depending on the clinical significance: set 4 including interactions with high and moderate clinical significance and set 5 with only highly clinically significant interactions as TP. The pAUROC results have shown that our similarity-based models performed better than PRR and *p*-values in the mentioned sets (Table 1). We also calculated precision in different top positions for all the methods including all the previous test sets (see S7 Table). Figs 4 and 5 show the precision versus the ranking for test sets 1 and 4, using all the levels of DDI knowledge in Drugdex and Drugs.com. Precision is improved when we used the similarity-based models to rank the DDIs.

## Discussion

**Fig 6:**
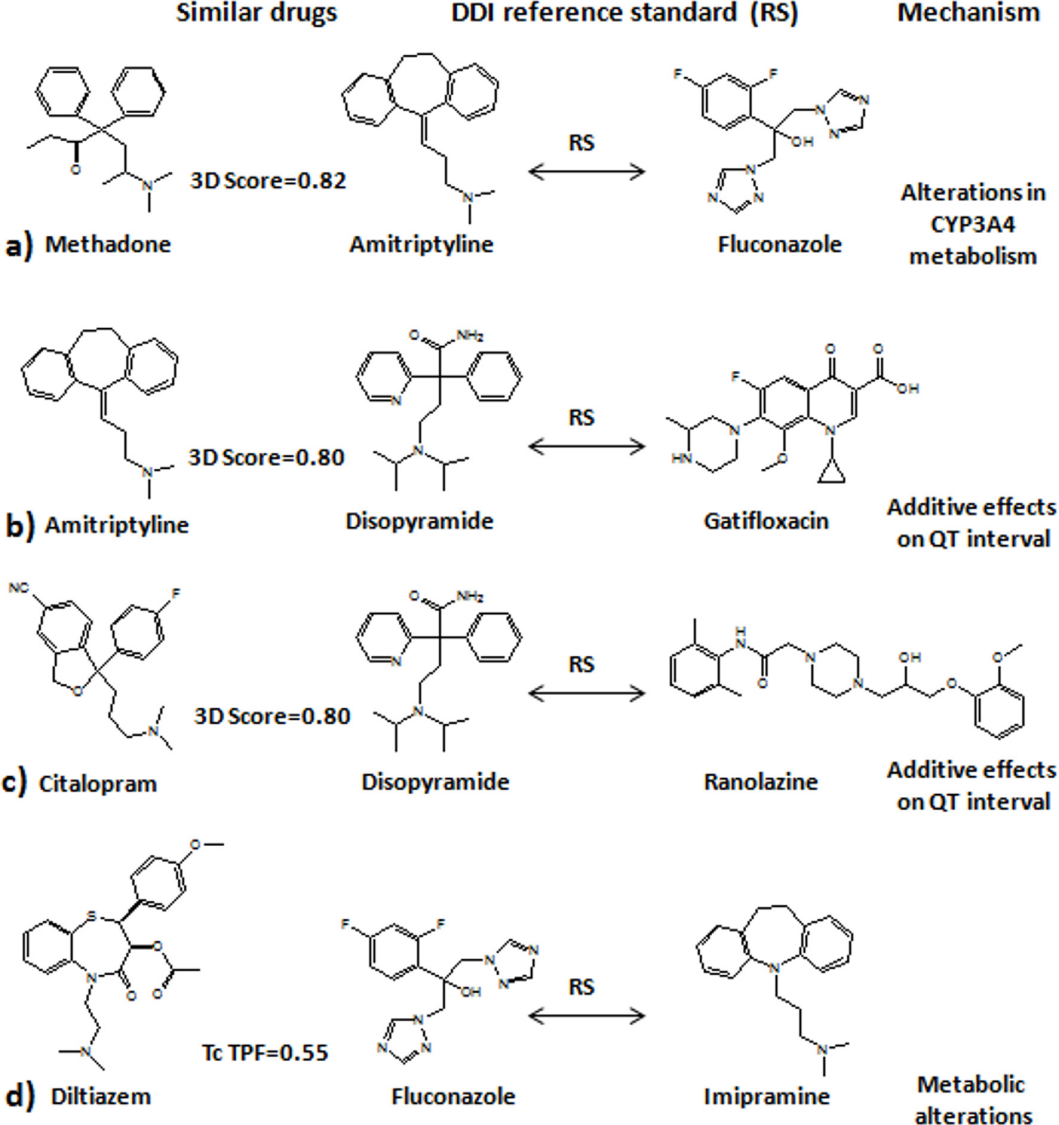
Examples of different pairs of similar drugs with different pharmacological profile detected by our models. Panel (a): methadone is similar to amitriptyline and predicted to interact with fluconazole (reference standard amitriptyline-fluconazole). Panel (b): amitriptyline is similar to disopyramide and predicted by the 3D model to interact with gatifloxacin (reference standard disopyramide—gatifloxacin). Panel (c): citalopram, was found to be similar to disopyramide and hence, to interact with ranolazine (reference standard disopyramide-ranolazine). Panel (d): diltiazem was found to be similar to fluconazole and predicted to interact with imipramine (reference standard fluconazole-imipramine).

The objective of our current study is to show the ability of cheminformatics to improve the analysis of DDI data extracted from a pharmacovigilance study. We applied different similarity-based models to improve DDI detection in the TWOSIDES database [18], an important source of DDI candidates extracted from FAERS [16]. Similarity models can be applied to other types of pharmacovigilance data, such as Electronic Medical Records, or claim databases [31]. These methods not only offer a possibility to improve the precision and hence, the detection of DDIs, but also provide additional information very useful in decision making. As an example, Table 2 shows some interactions detected by the different similarity models. For each DDI candidate, the method isolates the most similar drug pair in the reference standard, for which interaction information is available in the literature. This fact could be valuable for researchers to make decisions about the importance of the candidate, novelty or possible mechanism of action by which the drugs interact and cause the adverse effect. The DDI models can point out new DDI candidates based on the comparison of drugs that belong to the same pharmacological class. As an example in Table 2, the model generated the DDI candidate clarithromycin-verapamil because there is a similar interaction in our reference standard, the combination erythromycin-verapamil. In this case, both clarithromycin and erythromycin are macrolide antibiotics that belong to the same pharmacological class and can inhibit the CYP3A-mediated verapamil metabolism and increase verapamil exposure. However, the models are also able to detect some new candidates through the comparison of drugs in different classes. This is the case of some examples in Table 2 and Fig 6, such as the interaction between methadone and fluconazole that it is generated from the interaction amitriptyline-fluconazole. The model detected that the 3D structure of methadone, used in the treatment of opioid dependency and chronic pain, was similar to the tricyclic antidepressant amitriptyline (3D_score = 0.82). In both cases, fluconazole can decrease the CYP3A4 metabolism of amitriptyline and methadone and increase the serum concentration with a higher risk of causing drugs-related adverse effects, such as arrhythmias or QT interval prolongation [29]. Amitriptyline was also predicted by the 3D model to interact with gatifloxacin, an antibiotic of the fluoroquinolone family. The interaction was confirmed in Drugdex [29]. The model generated the candidate because amitriptyline was similar to the antiarrhythmic drug disopyramide (3D score = 0.80) (see Fig 6) and the interaction disopyramide and gatifloxacin was present in our reference standard. The probable mechanism of the interaction in both cases is due to additive effects on QT interval. A likely molecular mechanism of the drugs-QT prolongation is the blockade of the HERG potassium channel [32]. The selective serotonin reuptake inhibitor (SSRI) citalopram, was also found to be similar to disopyramide (3D score = 0.80) and hence, to interact with ranolazine. The combination disopyramide-ranolazine is associated with the risk of possible additive effects on QT prolongation. The same mechanism is predicted by the 3D model for the candidate citalopram-ranolazine and confirmed in Drugdex [29]. Another example described in our reference standard is the concomitant use of imipramine and fluconazole, associated with higher risk of QT prolongation due to possible alterations in imipramine metabolism. The Target model predicts the interaction between imipramine and diltiazem with the same mechanism associated. The probable mechanism described in Drugdex is in agreement and based on decreased imipramine clearance. Although in not all the cases the information about the adverse effect and mechanisms associated from the original DDI in the reference standard to the new candidate is correct, in many cases this information is valuable to assess the etiology and the importance of the DDI candidate.

**Table 2:** Example of some arrhythmia DDIs described in Drugdex and detected by the different similarity-based models (2D MACCS, 3D similarity, ADEPF, TPF, DDIPF and ATC) .

DDI candidates	Similar DDI in the initial reference standard	Models score	PRR
Verapamil-Clarithromycin	Erythromycin-Verapamil	Tc_2DMACCS = 0.98	5.47
Fluoxetine-Prochlorperazine	Thioridazine-Fluoxetine	Tc_2DMACCS = 0.81	2.79
Erythromycin-Prochlorperazine	Thioridazine- Erythromycin	Tc_2DMACCS = 0.81	5.50
Fluconazole-Methadone	Amitriptyline-Fluconazole	3D_score = 0.82	29.08
Gatifloxacin-Amitriptyline	Disopyramide-Gatifloxacin	3D_score = 0.80	8.30
Ranolazine-Citalopram	Disopyramide-Ranolazine	3D_score = 0.80	3.95
Trimipramine-Citalopram	Fluoxetine-Trimipramine	Tc_ADEPF = 0.42	5.89
Amiodarone-Ofloxacin	Moxifloxacin-Amiodarone	Tc_ADEPF = 0.39	3.31
Ziprasidone-Ofloxacin	Moxifloxacin-Ziprasidone	Tc_ADEPF = 0.39	4.37
Fluconazole-Quetiapine	Ziprasidone-Fluconazole	Tc_TPF = 0.84	2.53
Imipramine-Quetiapine	Ziprasidone-Imipramine	Tc_TPF = 0.84	6.27
Imipramine-Diltiazem	Fluconazole-Imipramine	Tc_TPF = 0.55	8.05
Gatifloxacin-Prochlorperazine	Perphenazine-Gatifloxacin	Tc_DDIPF = 0.94	7.78
Fluconazole-Doxepin	Amitriptyline-Fluconazole	Tc_DDIPF = 0.93	5.14
Gatifloxacin-Promethazine	Perphenazine-Gatifloxacin	Tc_DDIPF = 0.74	2.53
Ziprasidone-Azithromycin	Clarithromycin-Ziprasidone	Tc_ATC = 1.00	2.17
Quinidine-Azithromycin	Clarithromycin-Quinidine	Tc_ATC = 1.00	3.46
Fluoxetine-Nortriptyline	Trimipramine-Fluoxetine	Tc_ATC = 1.00	4.14

In the table we provided also proportional reporting ratio values (PRR) found in TWOSIDES data.

As we have shown previously, information provided by the different similarity scores can be implemented in the development of more complex models (PCA and LDA). Although the information is complementary, the different scoring measures showed some correlation. Table 3 shows the correlation coefficients between the six similarity measures implemented in this study.

**Table 3:** Correlation coefficients (r) between the six drug similarity measures.

	**ATC**	**2D MACCS**	**3D similarity**	**ADEPF**	**TPF**	**DDIPF**
**ATC**	1					
**2D MACCS**	0.32	1				
**3D similarity**	0.26	0.41	1			
**ADEPF**	0.15	0.09	0.02	1		
**TPF**	0.46	0.30	0.25	0.15	1	
**DDIPF**	0.41	0.28	0.22	0.23	0.32	1

Some knowledge measures, such as TPF and DDIPF, were more related to the therapeutic/pharmacological/chemical category defined in the ATC similarity (r = .46 and r = 0.41). This type of similarity measures are highly dependent on the information provided by knowledge databases that can be bias towards the pharmacological category. However, as shown in Fig 6, the similarity measures have the ability to capture intra-class and inter-class similarity. We calculated the number of drug pairs retrieved by the similarity scores in the different top percentile positions in a range of ATC classification, from zero (drugs in different class) to four ATC levels in common (drugs in the same class). All the measures showed a good recovery of the drug pairs in the same pharmacological class (4 coincident ATC levels). However, high similarity was also detected between some pairs of drugs with a totally different ATC classification (no ATC coincident levels). S8 Table shows the results of the analysis. In this article, similarity was integrated comparing drugs. However, additional similarity metrics could be added comparing adverse effects or adverse reactions caused by drugs combinations as a useful and alternative system to develop this type of predictor.

The test using Drugdex as a reference standard showed poor performance in sets 2 and 3, where only probable and well-established DDIs were considered positives. The similarity measures, capturing chemical and pharmacological features can detect with better precision DDIs deemed as theoretical by Drugdex (see set 1 with 115 theoretical, 39 probable and 10 well-established DDIs). The predictors are still useful pointing out possible dangerous drug combinations associated with severe outcomes. The test based on Drugs.com showed that the similarity models performed better than PRR and *p*-value scorings. In this set we used a DDI system classification based on clinical significance, related to the severity of the possible adverse events produced by the interactions.

Drug phenotypic, therapeutic, structural and genomic similarity modeling have also been applied to predict DDIs based on machine learning methods [33]. On the other hand, different types of similarity models were also previously published by our research group to predict different adverse effects [34] or new potential DDIs of different etiology [28]. In this study we showed the applicability of the similarity based models to improve the detection of DDIs that cause arrhythmias in pharmacovigilance data. Combining pharmacovigilance data with similarity modeling showed potential to facilitate the detection of new DDIs. In our study we integrated the data through an straightforward and simple approach that allows to obtain good performance values but also assists the researcher in the decision making process. The method allows the calculation and evaluation of new drugs in an external test set. Drugs in the test can be added to the matrix M_2_ providing similarity information between drugs in the test and drugs in the reference standard. The method will generate for the new drug-drug candidates in the test a score based on the maximum similarity against the set of DDIs in the reference standard. However, a limitation is that our method only predicts interactions between our 162 reference standard drugs and drugs in the test. No DDIs can be generated when both drugs implicated in the interaction are different from our 162 reference standard drugs. This fact limits the applicability of the developed models.

We applied similarity-based modeling to the DDI signals detected in FAERS when PRR>1 and p<.05. However, application of similarity modeling to all the DDIs included in the pharmacovigilance data could be an option to retrieve some interactions not detected by the data mining algorithm. This type of models could be implemented in the early detection of DDIs related to drugs newly introduced in the market and with not enough exposure in the population.

## Conclusions

In this study, we applied similarity-based modeling to the candidates selected through pharmacovigilance data mining of DDIs that can cause the ADE arrhythmia. When ranking the subset of DDI candidates, similarity-based modeling showed better performance than the parameters obtained in the pharmacovigilance data mining, such as PRR and *p*-values. The implementation of similarity-based modeling in pharmacovigilance improved precision of the final method and provided a mechanism for decision making. Our method is a useful tool as a pharmacovigilance resource that can help in the decision support of new DDIs.

## Supporting Information

S1 Fig Variance explained by each additional factor included in the PCA and AUROC results of LDA including from 1 to 5 variables in the model. (DOCX)

S1 Table Arrhythmia DDI reference standard (matrix M_1_) with 149 DDIs. (XLSX)

S2 Table Molecular structures (SMILES codes) for the drugs included in the study. (XLSX)

S3 Table Matrices M_2_ generated with the different similarity measures. (XLSX)

S4 Table List of DDIs generated in the matrices M_3_. (XLSX)

S5 Table List of 386 DDIs found in the intersection of TWOSIDES with the initial reference standard. (XLSX)

S6 Table pAUROCs using different methods to rank the 386 TWOSIDES candidates. (DOCX)

S7 Table Precision in different top positions ranking the TWOSIDES arrhythmia DDI candidates with different scoring methods (reference standard: Drugdex and Drugs.com). (DOCX)

S8 Table Number of drug pairs retrieved by the similarity scores in the different top percentile positions in a range of ATC classification, from zero (drugs in different class) to four ATC levels in common (drugs in the same class). (DOCX)
