# Supplementary material for: Improving Detection of Arrhythmia Drug-Drug Interactions in Pharmacovigilance Data through the Implementation of Similarity-Based Modeling
Source: PLoS One. 2015 Jun 12;10(6):e0129974. doi: 10.1371/journal.pone.0129974 (PMC4466327; doi:10.1371/journal.pone.0129974)

**Figure S1.** a) Percentage of the variance explained by each additional factor included in the PCA. b) AUROC results of LDA (14 positives and 372 negative cases) including from 1 to 5 variables in the model (inclusion cut-off: tolerance>.05, *F* to enter>1).


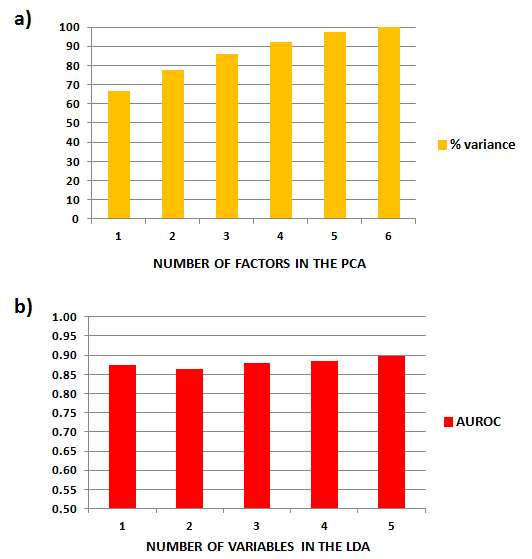

Supplement: S1 Fig — (DOCX) [file pone.0129974.s001.docx]
