# Supplementary material for: Improving Detection of Arrhythmia Drug-Drug Interactions in Pharmacovigilance Data through the Implementation of Similarity-Based Modeling
Source: PLoS One. 2015 Jun 12;10(6):e0129974. doi: 10.1371/journal.pone.0129974 (PMC4466327; doi:10.1371/journal.pone.0129974)
Supplement: S6 Table — (DOCX) [file pone.0129974.s007.docx]

**Supporting Information**

**Table S6.** pAUROCs using different methods to rank the 386 TWOSIDES candidates: PRR (Proportional Reporting Ratio), *p*-values, 2D structural similarity (MACCS), 3D structural similarity, ADEPF (Adverse Drug Effect Profile Fingerprint), TPF (Target Profile Fingerprint), DDIPF (Drug-Drug Interaction Profile Fingerprint), ATC-code fingerprint, PCA (Principal Component Analysis) and LDA (Linear Discriminant Analysis).

| Method used to rank the DDI candidates | pAUROCs (95% confidence interval) | *p*-level |
| --- | --- | --- |
| **PRR** | 0.62 (0.450 to 0.798) | 0.1628 |
| ***p*-value** | 0.67 (0.493 to 0.848) | 0.0601 |
| **2D MACCS** | 0.87 (0.768 to 0.964) | <0.0001 |
| **3D similarity** | 0.77 (0.643 to 0.906) | <0.0001 |
| **ADEPF** | 0.86 (0.809 to 0.909) | <0.0001 |
| **TPF** | 0.71 (0.580 to 0.838) | 0.0014 |
| **DDIPF** | 0.87 (0.768 to 0.978) | <0.0001 |
| **ATC-codes** | 0.84 (0.724 to 0.949) | <0.0001 |
| **PCA** | 0.87 (0.769 to 0.968) | <0.0001 |
| **LDA** | 0.90 (0.824 to 0.973) | <0.0001 |

pAUROCs calculated using the initial reference standard in TWOSIDES database (14 TP and 372 FP).
