# Supplementary material for: Improving Detection of Arrhythmia Drug-Drug Interactions in Pharmacovigilance Data through the Implementation of Similarity-Based Modeling
Source: PLoS One. 2015 Jun 12;10(6):e0129974. doi: 10.1371/journal.pone.0129974 (PMC4466327; doi:10.1371/journal.pone.0129974)
Supplement: S7 Table — (DOCX) [file pone.0129974.s008.docx]

**Supporting Information**

**Table S7.** Precision in different top positions ranking the TWOSIDES arrhythmia DDI candidates with different scoring methods: PRR (Proportional Reporting Ratio), *p*-values, 2D structural similarity (MACCS), 3D structural similarity, ADEPF (Adverse Drug Effect Profile Fingerprint), TPF (Target Profile Fingerprint), DDIPF (Drug-Drug Interaction Profile Fingerprint), ATC-code fingerprint, PCA (Principal Component Analysis) and LDA (Linear Discriminant Analysis).

In set 1, interactions well-established, probable and theoretical are considered true positives (TP). In set 2, interactions well-established and probable are considered TP. In set 3, only interactions well-established are considered TP. Set 4 included interactions with high and moderate clinical significance as TP. Set 5 included only highly clinically significant interactions as TP.

| Precision using Drugdex Reference Standard  Test set 1: interactions well-established, probable and theoretical | | | | | | | | | | |
| --- | --- | --- | --- | --- | --- | --- | --- | --- | --- | --- |
| TOP position | PRR | *p*-values | 2D MACCS | 3D similarity | ADEPF | TPF | DDIPF | ATC-code | PCA | LDA |
| 10 | 0.50 | 0.50 | 0.90 | 1.00 | 0.70 | 0.70 | 0.90 | 0.80 | 1.00 | 1.00 |
| 20 | 0.55 | 0.70 | 0.85 | 0.85 | 0.75 | 0.75 | 0.90 | 0.80 | 0.80 | 0.85 |
| 30 | 0.60 | 0.63 | 0.77 | 0.87 | 0.77 | 0.70 | 0.87 | 0.80 | 0.87 | 0.80 |
| 40 | 0.60 | 0.53 | 0.75 | 0.88 | 0.83 | 0.60 | 0.78 | 0.75 | 0.83 | 0.80 |
| 50 | 0.52 | 0.46 | 0.70 | 0.80 | 0.78 | 0.52 | 0.78 | 0.76 | 0.80 | 0.80 |
| 60 | 0.48 | 0.40 | 0.68 | 0.77 | 0.75 | 0.55 | 0.77 | 0.78 | 0.80 | 0.77 |
| 70 | 0.44 | 0.41 | 0.69 | 0.76 | 0.73 | 0.60 | 0.77 | 0.76 | 0.79 | 0.74 |
| 80 | 0.45 | 0.41 | 0.65 | 0.74 | 0.73 | 0.65 | 0.78 | 0.76 | 0.79 | 0.73 |
| 90 | 0.46 | 0.43 | 0.63 | 0.72 | 0.73 | 0.64 | 0.76 | 0.73 | 0.78 | 0.73 |
| 100 | 0.41 | 0.43 | 0.64 | 0.68 | 0.71 | 0.64 | 0.74 | 0.70 | 0.78 | 0.71 |
|  |  |  |  |  |  |  |  |  |  |  |
| Precision using Drugdex Reference Standard  Test set 2: interactions well-established and probable | | | | | | | | | | |
| TOP position | PRR | *p*-values | 2D MACCS | 3D similarity | ADEPF | TPF | DDIPF | ATC-code | PCA | LDA |
| 10 | 0.20 | 0.10 | 0.10 | 0.10 | 0.10 | 0.00 | 0.30 | 0.30 | 0.20 | 0.30 |
| 20 | 0.20 | 0.05 | 0.10 | 0.10 | 0.05 | 0.00 | 0.20 | 0.15 | 0.10 | 0.25 |
| 30 | 0.20 | 0.07 | 0.10 | 0.10 | 0.10 | 0.03 | 0.13 | 0.10 | 0.10 | 0.17 |
| 40 | 0.15 | 0.08 | 0.15 | 0.13 | 0.13 | 0.10 | 0.10 | 0.15 | 0.10 | 0.15 |
| 50 | 0.14 | 0.08 | 0.14 | 0.10 | 0.16 | 0.10 | 0.10 | 0.12 | 0.10 | 0.18 |
| 60 | 0.13 | 0.07 | 0.17 | 0.08 | 0.13 | 0.13 | 0.12 | 0.12 | 0.13 | 0.15 |
| 70 | 0.11 | 0.10 | 0.14 | 0.10 | 0.13 | 0.11 | 0.13 | 0.13 | 0.13 | 0.16 |
| 80 | 0.14 | 0.13 | 0.15 | 0.10 | 0.11 | 0.13 | 0.15 | 0.18 | 0.14 | 0.15 |
| 90 | 0.13 | 0.13 | 0.14 | 0.11 | 0.14 | 0.11 | 0.16 | 0.17 | 0.14 | 0.19 |
| 100 | 0.12 | 0.14 | 0.14 | 0.12 | 0.15 | 0.12 | 0.15 | 0.17 | 0.15 | 0.18 |
|  |  |  |  |  |  |  |  |  |  |  |
| Precision using Drugdex Reference Standard  Test set 3: interactions well-established | | | | | | | | | | |
| TOP position | PRR | *p*-values | 2D MACCS | 3D similarity | ADEPF | TPF | DDIPF | ATC-code | PCA | LDA |
| 10 | 0.10 | 0.00 | 0.10 | 0.00 | 0.00 | 0.00 | 0.10 | 0.10 | 0.10 | 0.10 |
| 20 | 0.05 | 0.00 | 0.05 | 0.00 | 0.00 | 0.00 | 0.10 | 0.05 | 0.05 | 0.10 |
| 30 | 0.03 | 0.00 | 0.03 | 0.00 | 0.00 | 0.00 | 0.07 | 0.03 | 0.03 | 0.07 |
| 40 | 0.03 | 0.00 | 0.03 | 0.03 | 0.00 | 0.00 | 0.05 | 0.03 | 0.03 | 0.05 |
| 50 | 0.02 | 0.00 | 0.02 | 0.02 | 0.00 | 0.00 | 0.04 | 0.02 | 0.02 | 0.06 |
| 60 | 0.02 | 0.00 | 0.02 | 0.02 | 0.00 | 0.00 | 0.03 | 0.02 | 0.03 | 0.05 |
| 70 | 0.01 | 0.01 | 0.01 | 0.01 | 0.00 | 0.00 | 0.03 | 0.03 | 0.03 | 0.06 |
| 80 | 0.01 | 0.03 | 0.04 | 0.01 | 0.00 | 0.00 | 0.03 | 0.08 | 0.03 | 0.06 |
| 90 | 0.01 | 0.02 | 0.04 | 0.02 | 0.01 | 0.00 | 0.02 | 0.07 | 0.02 | 0.08 |
| 100 | 0.01 | 0.02 | 0.04 | 0.02 | 0.01 | 0.01 | 0.03 | 0.06 | 0.02 | 0.07 |
|  |  |  |  |  |  |  |  |  |  |  |
| Precision using Drugs.com Reference Standard  Test set 4: interactions with high and moderate clinical significance | | | | | | | | | | |
| TOP position | PRR | *p*-values | 2D MACCS | 3D similarity | ADEPF | TPF | DDIPF | ATC-code | PCA | LDA |
| 10 | 0.80 | 0.80 | 0.90 | 1.00 | 0.90 | 1.00 | 1.00 | 0.80 | 1.00 | 1.00 |
| 20 | 0.75 | 0.90 | 0.90 | 0.95 | 0.90 | 0.95 | 0.95 | 0.85 | 0.90 | 0.90 |
| 30 | 0.73 | 0.83 | 0.93 | 0.97 | 0.90 | 0.87 | 0.90 | 0.87 | 0.93 | 0.87 |
| 40 | 0.80 | 0.73 | 0.90 | 0.98 | 0.93 | 0.78 | 0.90 | 0.83 | 0.90 | 0.85 |
| 50 | 0.78 | 0.70 | 0.82 | 0.88 | 0.86 | 0.72 | 0.88 | 0.84 | 0.88 | 0.84 |
| 60 | 0.70 | 0.67 | 0.83 | 0.87 | 0.83 | 0.72 | 0.88 | 0.85 | 0.88 | 0.82 |
| 70 | 0.67 | 0.70 | 0.81 | 0.86 | 0.80 | 0.74 | 0.89 | 0.84 | 0.89 | 0.83 |
| 80 | 0.65 | 0.69 | 0.76 | 0.84 | 0.79 | 0.78 | 0.89 | 0.81 | 0.90 | 0.83 |
| 90 | 0.67 | 0.69 | 0.77 | 0.84 | 0.80 | 0.76 | 0.87 | 0.80 | 0.91 | 0.83 |
| 100 | 0.64 | 0.66 | 0.77 | 0.84 | 0.80 | 0.77 | 0.86 | 0.78 | 0.91 | 0.81 |
|  |  |  |  |  |  |  |  |  |  |  |
| Precision using Drugs.com Reference Standard  Test set 5: interactions with high clinical significance | | | | | | | | | | |
| TOP position | PRR | *p*-values | 2D MACCS | 3D similarity | ADEPF | TPF | DDIPF | ATC-code | PCA | LDA |
| 10 | 0.30 | 0.50 | 0.80 | 0.80 | 0.40 | 0.40 | 0.60 | 0.60 | 0.60 | 0.70 |
| 20 | 0.45 | 0.55 | 0.55 | 0.60 | 0.40 | 0.30 | 0.40 | 0.70 | 0.45 | 0.55 |
| 30 | 0.43 | 0.50 | 0.50 | 0.60 | 0.47 | 0.37 | 0.40 | 0.57 | 0.50 | 0.50 |
| 40 | 0.43 | 0.43 | 0.48 | 0.55 | 0.50 | 0.35 | 0.33 | 0.48 | 0.48 | 0.48 |
| 50 | 0.42 | 0.36 | 0.40 | 0.44 | 0.42 | 0.30 | 0.34 | 0.46 | 0.42 | 0.48 |
| 60 | 0.38 | 0.33 | 0.37 | 0.40 | 0.37 | 0.32 | 0.40 | 0.43 | 0.43 | 0.45 |
| 70 | 0.33 | 0.31 | 0.36 | 0.39 | 0.37 | 0.39 | 0.41 | 0.44 | 0.43 | 0.46 |
| 80 | 0.33 | 0.29 | 0.34 | 0.38 | 0.36 | 0.35 | 0.44 | 0.43 | 0.45 | 0.41 |
| 90 | 0.32 | 0.29 | 0.32 | 0.37 | 0.39 | 0.37 | 0.41 | 0.41 | 0.46 | 0.39 |
| 100 | 0.29 | 0.27 | 0.34 | 0.38 | 0.38 | 0.36 | 0.39 | 0.39 | 0.47 | 0.37 |
