# Supplementary material for: Improving Detection of Arrhythmia Drug-Drug Interactions in Pharmacovigilance Data through the Implementation of Similarity-Based Modeling
Source: PLoS One. 2015 Jun 12;10(6):e0129974. doi: 10.1371/journal.pone.0129974 (PMC4466327; doi:10.1371/journal.pone.0129974)
Supplement: S8 Table — (DOCX) [file pone.0129974.s009.docx]

**Supporting Information**

**Table S8.** Number of drug pairs retrieved by the similarity scores (2D MACCS structural similarity, 3D structural similarity, adverse effect similarity, target similarity and drug-drug interaction similarity) in the different top percentile positions in a range of ATC classification, from zero (drugs in different class) to four ATC levels in common (drugs in the same class).

| Number of drug pairs retrieved by the similarity scores | | | | | |
| --- | --- | --- | --- | --- | --- |
| 2D MACCS similarity | | | | | |
|  | No ATC codes in common | 1 ATC codes in common | 2 ATC codes in common | 3 ATC codes in common | 4 ATC codes in common |
| x≥Top10% | 771 | 160 | 73 | 135 | 165 |
| Top10%>x≥Top20% | 994 | 161 | 36 | 86 | 27 |
| Top20%>x≥Top30% | 1012 | 188 | 29 | 59 | 16 |
| Top30%>x≥Top40% | 1041 | 175 | 30 | 48 | 10 |
| Top40%>x≥Top50% | 1089 | 165 | 18 | 28 | 4 |
| Top50%>x≥Top60% | 1090 | 168 | 11 | 32 | 3 |
| Top60%>x≥Top70% | 1110 | 155 | 13 | 22 | 4 |
| Top70%>x≥Top80% | 1121 | 150 | 13 | 16 | 4 |
| Top80%>x≥Top90% | 1096 | 173 | 13 | 21 | 1 |
| Top90%>x≥Top100% | 1095 | 176 | 18 | 15 | 1 |
|  |  |  |  |  |  |
| 3D similarity | | | | | |
|  | No ATC codes in common | 1 ATC codes in common | 2 ATC codes in common | 3 ATC codes in common | 4 ATC codes in common |
| x≥Top10% | 639 | 306 | 37 | 179 | 143 |
| Top10%>x≥Top20% | 989 | 206 | 24 | 68 | 17 |
| Top20%>x≥Top30% | 1033 | 185 | 26 | 45 | 15 |
| Top30%>x≥Top40% | 1067 | 162 | 25 | 37 | 13 |
| Top40%>x≥Top50% | 1105 | 139 | 18 | 32 | 10 |
| Top50%>x≥Top60% | 1087 | 146 | 8 | 50 | 13 |
| Top60%>x≥Top70% | 1123 | 134 | 13 | 19 | 15 |
| Top70%>x≥Top80% | 1144 | 114 | 31 | 10 | 5 |
| Top80%>x≥Top90% | 1095 | 153 | 48 | 5 | 3 |
| Top90%>x≥Top100% | 1137 | 126 | 24 | 17 | 1 |
|  |  |  |  |  |  |
| Adverse effect profile similarity | | | | | |
|  | No ATC codes in common | 1 ATC codes in common | 2 ATC codes in common | 3 ATC codes in common | 4 ATC codes in common |
| x≥Top10% | 863 | 224 | 37 | 109 | 71 |
| Top10%>x≥Top20% | 1012 | 192 | 28 | 55 | 17 |
| Top20%>x≥Top30% | 1064 | 181 | 11 | 39 | 9 |
| Top30%>x≥Top40% | 1113 | 151 | 11 | 24 | 5 |
| Top40%>x≥Top50% | 972 | 172 | 21 | 66 | 73 |
| Top50%>x≥Top60% | 907 | 236 | 40 | 89 | 32 |
| Top60%>x≥Top70% | 1102 | 134 | 25 | 30 | 13 |
| Top70%>x≥Top80% | 1134 | 116 | 13 | 36 | 5 |
| Top80%>x≥Top90% | 1134 | 127 | 21 | 13 | 9 |
| Top90%>x≥Top100% | 1118 | 138 | 47 | 1 | 1 |
|  |  |  |  |  |  |
| Target profile similarity | | | | | |
|  | No ATC codes in common | 1 ATC codes in common | 2 ATC codes in common | 3 ATC codes in common | 4 ATC codes in common |
| x≥Top10% | 622 | 250 | 55 | 217 | 160 |
| Top10%>x≥Top20% | 842 | 249 | 32 | 141 | 40 |
| Top20%>x≥Top30% | 992 | 235 | 30 | 35 | 12 |
| Top30%>x≥Top40% | 1087 | 160 | 32 | 22 | 3 |
| Top40%>x≥Top50% | 1141 | 141 | 11 | 10 | 1 |
| Top50%>x≥Top60% | 1168 | 110 | 10 | 8 | 8 |
| Top60%>x≥Top70% | 1111 | 165 | 15 | 11 | 2 |
| Top70%>x≥Top80% | 1183 | 102 | 8 | 7 | 4 |
| Top80%>x≥Top90% | 1145 | 132 | 14 | 10 | 3 |
| Top90%>x≥Top100% | 1128 | 127 | 47 | 1 | 2 |
|  |  |  |  |  |  |
| Drug-drug interaction profile similarity | | | | | |
|  | No ATC codes in common | 1 ATC codes in common | 2 ATC codes in common | 3 ATC codes in common | 4 ATC codes in common |
| x≥Top10% | 727 | 209 | 54 | 130 | 184 |
| Top10%>x≥Top20% | 892 | 217 | 44 | 131 | 20 |
| Top20%>x≥Top30% | 1059 | 176 | 13 | 44 | 12 |
| Top30%>x≥Top40% | 1086 | 156 | 21 | 35 | 6 |
| Top40%>x≥Top50% | 1058 | 162 | 33 | 48 | 3 |
| Top50%>x≥Top60% | 1125 | 132 | 23 | 21 | 3 |
| Top60%>x≥Top70% | 1094 | 168 | 14 | 23 | 5 |
| Top70%>x≥Top80% | 1080 | 179 | 20 | 24 | 1 |
| Top80%>x≥Top90% | 1167 | 125 | 7 | 4 | 1 |
| Top90%>x≥Top100% | 1131 | 147 | 25 | 2 | 0 |
